# Is it ethical and safe to use non-invasive brain stimulation as a cognitive and motor enhancer device for military services? A reply to Sehm and Ragert (2013)

**DOI:** 10.3389/fnhum.2013.00874

**Published:** 2013-12-16

**Authors:** Jerome Brunelin, Jean Levasseur-Moreau, Shirley Fecteau

**Affiliations:** ^1^Centre Interdisciplinaire de Recherche en Réadaptation et en Intégration Sociale de l'Université Laval, Centre de Recherche de l'Institut Universitaire en Santé Mentale de Québec, Faculté de Médecine, Université LavalQuebec, QC, Canada; ^2^Centre Hospitalier le Vinatier, Université de Lyon, F-69003, Université Claude Bernard Lyon I, EA 4615Bron, Lyon, France; ^3^Berenson-Allen Center for Noninvasive Brain Stimulation, Beth Israel Deaconess Medical Center, Harvard Medical SchoolBoston, MA, USA

**Keywords:** non-invasive brain stimulation, motor function, cognitive function, security, transcranial direct current stimulation (tDCS), neuroenhancement, transcranial magnetic stimulation (TMS)

We appreciate the comment from Sehm and Ragert (2013) on our review article published in the Frontiers Research Topic on *Neuroscience perspectives on Security* (Levasseur-Moreau et al., 2013) and we want to clearly and briefly reaffirm our position to avoid misinterpretations.

The goal of our article was to review data suggesting that non-invasive brain stimulation (NIBS) can enhance performance in healthy volunteers (in line with our expertise) and to focus on motor and cognitive functions that are relevant for security purposes (in line with the Research Topic). We did not take position on whether or not NIBS may eventually serve security because we believe that there are ethics and safety aspects to be studied before considering NIBS as a neuroenhancer device for healthy individuals.

The goal of our review paper was not to examine the ethics and safety of NIBS. We did call for an open and fair debate on the ethics and safety of using NIBS in healthy volunteers because we consider it is our responsibility to at least acknowledge these aspects although they were beyond the scope of our paper. We believe that review articles (as our) should not be considered as encouragement to an irresponsible use of NIBS and we therefore, thank Sehm and Ragert for taking part in this debate.

As neuroscientists we cannot ignore data suggesting to some extent that NIBS might eventually be used as a cognitive enhancer and it is our obligation to discuss their limitations in terms of safety, ethics, transferability, and meaningfulness as we did in Levasseur-Moreau et al. (2013). We thus, raised potential safety and ethical concerns of using NIBS in healthy participants and we referred readers to articles specifically addressing these major questions (e.g., Illes and Bird, 2006; Forlini et al., 2013).

This debate on ethics and safety on the use of NIBS for cognitive enhancement should certainly be pursued among scientists (e.g., Bikson et al., 2013) but we should also seek participation of policy makers, ethicists and manufacturers, since, whether we like it or not, there is a fast-growing market promoting do-it-yourself brain stimulation devices proposing NIBS for a recreational use, especially transcranial Direct Current Stimulation (tDCS). We thus, welcome the effort of policy makers such as The California Department of Public Health (CDPH) that “warned consumers not to use the unapproved medical device sold on the Internet as a tDCS Home Device Kit” (see http://www.cdph.ca.gov/Pages/NR13-029.aspx). As already stated in Levasseur-Moreau et al. (2013), we believe that this discussion should also encompass all potential nonpharmacologic neuroenhancers and brain-boosting drugs that can improve performance of healthy individuals including military personnel.

If our position on the ethics or safety in the use of NIBS in our article has been misinterpreted or is unclear, we hereby want to reaffirm it because these topics strongly matter to us: in our opinion, benefits and risks in terms of ethics and safety must be clearly weighed before any use of NIBS as a cognitive enhancer in healthy population. NIBS protocols must be reviewed by independent and competent institutional review boards. Stimulation sessions should be delivered by adequately trained staff in a secure environment (e.g., hospital setting) and with strict inclusion and exclusion criteria to ensure safety of participants in accordance with international guidelines (for instance, see Rossi et al., 2009).
